# Supplementary material for: Adherence to Mediterranean diet and female urinary incontinence: Evidence from the NHANES database
Source: PLoS One. 2024 Oct 22;19(10):e0311771. doi: 10.1371/journal.pone.0311771 (PMC11495614; doi:10.1371/journal.pone.0311771)
Supplement: S1 Table — (DOCX) [file pone.0311771.s001.docx]

Table S1. Sensitivity analyses for data before and after imputation

| Variables | Before imputation | After imputation | Statistics | *P* |
| --- | --- | --- | --- | --- |
| Education level, n (%) |  |  | χ^2^=4.81 | 0.090 |
| Less than high school | 3385 (16.65) | 3392 (16.66) |  |  |
| High school | 3095 (24.62) | 3099 (24.62) |  |  |
| More than high school | 6798 (58.73) | 6800 (58.71) |  |  |
| Marital status, n (%) |  |  | χ^2^=3.21 | 0.200 |
| Married | 6705 (57.65) | 6708 (57.65) |  |  |
| Never married | 1370 (8.15) | 1373 (8.17) |  |  |
| Others | 5210 (34.20) | 5210 (34.18) |  |  |
| Smoking, n (%) |  |  | χ^2^=1.70 | 0.193 |
| No | 8262 (59.11) | 8264 (59.08) |  |  |
| Yes | 5024 (40.89) | 5027 (40.92) |  |  |
| Drinking, n (%) |  |  | χ^2^=2.85 | 0.091 |
| No | 5134 (31.10) | 5212 (30.97) |  |  |
| Yes | 7889 (68.90) | 8079 (69.03) |  |  |
| Status of menopause, n (%) |  |  | χ^2^=2.15 | 0.142 |
| No | 5448 (42.59) | 5448 (42.56) |  |  |
| Yes | 7837 (57.41) | 7843 (57.44) |  |  |
| Depression, n (%) |  |  | χ^2^=3.13 | 0.077 |
| No | 11656 (89.73) | 11771 (89.68) |  |  |
| Yes | 1496 (10.27) | 1520 (10.32) |  |  |
| BMI, kg/m^2^, Mean (S.E) | 29.48 (0.12) | 29.48 (0.12) | t=-1.10 | 0.273 |
| NLR, Mean (S.E) | 2.14 (0.02) | 2.14 (0.02) | t=-0.42 | 0.672 |

S.E: standard error; χ^2^: chi-square test; t: t-test.

BMI: body mass index; NLR: neutrophil to lymphocyte ratio.
