# Supplementary material for: Adherence to Mediterranean diet and female urinary incontinence: Evidence from the NHANES database
Source: PLoS One. 2024 Oct 22;19(10):e0311771. doi: 10.1371/journal.pone.0311771 (PMC11495614; doi:10.1371/journal.pone.0311771)
Supplement: Attachment — Submitted filename: Response to reviewers.docx [file pone.0311771.s002.docx]

**Review Comments to the Author**

Please use the space provided to explain your answers to the questions above. You may also include additional comments for the author, including concerns about dual publication, research ethics, or publication ethics. (Please upload your review as an attachment if it exceeds 20,000 characters)

Reviewer #1: The presented knowledge is not novel. However the conducted study demonstrates high scientific quality and is both necessary and relevant to the advancement of nutritional science.

1. The introduction is correctly presented, with the Authors fully justifying the need for such an analysis using the numerous studies available on the subject.

Reply: Thanks for your recognition for our study.
2. The study was conducted without methodological errors. The methods are described in detail and it is possible to replicate the study.

Reply: Thanks for your recognition for our study.
3. The description of the results is correct and in line with the usual description of manuscripts. The tables and figures presented are done correctly.

Reply: Thanks for your recognition for our study.
4. The Discussion section contained an accurate reference of the results obtained to the studies of other authors. However, I believe that other physiological factors that may significantly influence the condition under study have been discussed too generally. A broader discussion of the topic would definitely increase the scientific value of this paper.

Reply: Thanks for your comment. According to your opinions, we checked our manuscript and the relevant physiological mechanisms were described in further detail. All changes were marked in red in the manuscript.
5. The conclusions are well formulated and fully relate to the results obtained.

Reply: Thanks for your recognition for our study.
6. The formatting of the text and the layout of the manuscript are correct, according to the requirements of the journal.

Reply: Thanks for your recognition for our study.

Reviewer #2: Xie et al. have performed a study on the adherence to Mediterranean diet and female urinary incontinence using the NHANES data from 2005 to 2018. The study findings are interesting and the manuscript is well-written. These are my comments:
- Authors should add strengths to their manuscript before the limitation.

Reply: Thanks for your comment. We have supplemented the strengths of our study in the Discussion section.
- A paragraph summarizing the clinical take-home message of this manuscript should be added to the discussion.

Reply: Thanks for your comment. We have supplemented the clinical take-home information in the Discussion section.
- The references prior to 2010 could be updated with those after 2010 since they provide more up-to-date findings.

Reply: Thanks for your comment. We have updated the references prior to 2010 in the manuscript. However, there were still several studies prior to 2010 that were very relevant to our study, so we did not replace these references.
